# Age-related brain deviations and aggression

**DOI:** 10.1017/S003329172200068X

**Published:** 2023-07

**Authors:** Nathalie E. Holz, Dorothea L. Floris, Alberto Llera, Pascal M. Aggensteiner, Seyed Mostafa Kia, Thomas Wolfers, Sarah Baumeister, Boris Böttinger, Jeffrey C. Glennon, Pieter J. Hoekstra, Andrea Dietrich, Melanie C. Saam, Ulrike M. E. Schulze, David J. Lythgoe, Steve C. R. Williams, Paramala Santosh, Mireia Rosa-Justicia, Nuria Bargallo, Josefina Castro-Fornieles, Celso Arango, Maria J. Penzol, Susanne Walitza, Andreas Meyer-Lindenberg, Marcel Zwiers, Barbara Franke, Jan Buitelaar, Jilly Naaijen, Daniel Brandeis, Christian Beckmann, Tobias Banaschewski, Andre F. Marquand

**Affiliations:** 1 Department of Child and Adolescent Psychiatry and Psychotherapy, Central Institute of Mental Health, Medical Faculty Mannheim/Heidelberg University, Mannheim, Germany; 2 Donders Center for Brain, Cognition and Behavior, Radboud University Nijmegen, Nijmegen, The Netherlands; 3 Department for Cognitive Neuroscience, Radboud University Medical Center Nijmegen, Nijmegen, The Netherlands; 4 Methods of Plasticity Research, Department of Psychology, University of Zurich, Zurich, Switzerland; 5 NORMENT, KG Jebsen Centre for Psychosis Research, Division of Mental Health and Addiction, Oslo University Hospital & Institute of Clinical Medicine, University of Oslo, Oslo, Norway; 6 Department of Child and Adolescent Psychiatry, University of Groningen, University Medical Center Groningen, Groningen, The Netherlands; 7 Department of Child and Adolescent Psychiatry/Psychotherapy, University Hospital, University of Ulm, Ulm, Germany; 8 Department of Neuroimaging, Institute of Psychiatry, Psychology & Neuroscience, King's College London, London, UK; 9 Department of Child Psychiatry, Institute of Psychiatry, Psychology & Neuroscience, King's College London, London, UK; 10 Centre for Interventional Paediatric Psychopharmacology and Rare Diseases (CIPPRD), South London and Maudsley NHS Trust, London, UK; 11 Clinic Image Diagnostic Center (CDIC), Hospital Clinic of Barcelona; Magnetic Resonance Image Core Facility, IDIBAPS, Barcelona, Spain; 12 Child and Adolescent Psychiatry and Psychology Department, Institute Clinic of Neurosciences, Hospital Clinic of Barcelona, IDIBAPS, Barcelona, Spain; 13 Child and Adolescent Psychiatry and Psychology Department, Department of Medicine, 2017SGR881, Institute Clinic of Neurosciences, Hospital Clinic of Barcelona, CIBERSAM, IDIBAPS, University of Barcelona, Barcelona, Spain; 14 Child and Adolescent Psychiatry Department, Institute of Psychiatry and Mental health, Hospital General Universitario Gregorio Marañón School of Medicine, Universidad Complutense, IiSGM, CIBERSAM, Madrid, Spain; 15 Department of Child and Adolescent Psychiatry and Psychotherapy, Psychiatric Hospital, University of Zurich, Zurich, Switzerland; 16 Department of Psychiatry and Psychotherapy, Central Institute of Mental Health, Medical Faculty Mannheim/Heidelberg University, Mannheim, Germany; 17 Department of Human Genetics, Radboud University Medical Center, Nijmegen, The Netherlands; 18 Department of Psychiatry, Radboud University Medical Center, Nijmegen, The Netherlands; 19 Karakter Child and Adolescent Psychiatry University Center, Nijmegen, The Netherlands; 20 Center for Integrative Human Physiology, University of Zurich, Zurich, Switzerland; 21 Neuroscience Center Zurich, University of Zurich and ETH Zurich, Zurich, Switzerland; 22 Centre for Functional MRI of the Brain, University of Oxford, Oxford, UK

**Keywords:** Aggression, disruptive behavior disorders, emotion processing, fMRI, normative modeling

## Abstract

**Background:**

Disruptive behavior disorders (DBD) are heterogeneous at the clinical and the biological level. Therefore, the aims were to dissect the heterogeneous neurodevelopmental deviations of the affective brain circuitry and provide an integration of these differences across modalities.

**Methods:**

We combined two novel approaches. First, normative modeling to map deviations from the typical age-related pattern at the level of the individual of (i) activity during emotion matching and (ii) of anatomical images derived from DBD cases (*n* = 77) and controls (*n* = 52) aged 8–18 years from the EU-funded Aggressotype and MATRICS consortia. Second, linked independent component analysis to integrate subject-specific deviations from both modalities.

**Results:**

While cases exhibited on average a higher activity than would be expected for their age during face processing in regions such as the amygdala when compared to controls these positive deviations were widespread at the individual level. A multimodal integration of all functional and anatomical deviations explained 23% of the variance in the clinical DBD phenotype. Most notably, the top marker, encompassing the default mode network (DMN) and subcortical regions such as the amygdala and the striatum, was related to aggression across the whole sample.

**Conclusions:**

Overall increased age-related deviations in the amygdala in DBD suggest a maturational delay, which has to be further validated in future studies. Further, the integration of individual deviation patterns from multiple imaging modalities allowed to dissect some of the heterogeneity of DBD and identified the DMN, the striatum and the amygdala as neural signatures that were associated with aggression.

## Introduction

Aggressive behavior is commonly seen in disruptive behavior disorders (DBD), including oppositional defiant disorder (ODD) and conduct disorder (CD), prevalent in 5.7% of the youth population (Polanczyk, Salum, Sugaya, Caye, & Rohde, 2015). Given frequent comorbidity, both disorders have been conceptualized on a continuum with CD being more severe regarding the presentation of aggressive symptoms (Pardini, Frick, & Moffitt, 2010). Research investigating the neurobiology of DBD has highlighted their complex and multifaceted nature, reflected both in altered brain structure and function. In particular, regions that support affective processing (Kohls et al., 2020) and executive functions have been shown to be disrupted in DBD (Blair, Veroude, & Buitelaar, 2018; Fairchild et al., 2019). As such, recent meta-analyses provided evidence for functional differences in the insula, the fusiform gyrus and top-down control regions such as the medial frontal gyrus and the dorsolateral prefrontal cortex during emotion processing in DBD when compared to healthy controls (Alegria, Radua, & Rubia, 2016; Noordermeer, Luman, & Oosterlaan, 2016). Notably, highly heterogeneous results exist for the amygdala with hyperactivity and hypoactivity observed depending on the aggression subtype (Aggensteiner et al., 2020; Lozier, Cardinale, VanMeter, & Marsh, 2014). In line with this, inconsistencies also exist between results of meta-analyses, where differences in the amygdala have been detected in one meta-analysis (Noordermeer et al., 2016), but not in another (Alegria et al., 2016). In addition to these functional alterations, structural differences have been detected in a meta-analysis indicating reduced volumes in similar regions in youths with conduct problems, i.e. the insula, the medial frontal gyrus, the fusiform gyrus, and the amygdala (Rogers & De Brito, 2016). By analogy to functional findings, some abnormalities in volume also seem to be influenced by the presence of callous-unemotional (CU) traits (Rogers & De Brito, 2016). In general, a good understanding of brain development in DBD is largely lacking. This is despite evidence for a strong neurodevelopmental component, especially for early-onset DBD, implicating an origin in infancy or childhood, atypical brain development, neurocognitive deficits and a life-course-persistent manifestation (Raine, 2018).

Overall, recent attempts to uncover the neurobiology of DBD have largely focused on investigating subgroups of patients (Blair, Leibenluft, & Pine, 2015) based on biology and/or on symptoms. However, especially regarding CD, highly heterogeneous symptom profiles qualify for a diagnosis (Fairchild et al., 2019; Viding & McCrory, 2020). The dominant case–control analysis approach provides inferences about the ‘average patient’ of a particular group and by definition does not provide information on inter-individual differences within each grouping but rather considers these as noise. Finally, as mentioned above, DBD has been shown to affect multiple quantitative phenotypes rather than single features, suggesting that integration of different imaging modalities would allow for a more refined, comprehensive neural characterization of DBD.

Therefore, the aim of this study was to understand individual neurobiological variations beyond group-level differences by investigating functional and structural imaging data via a normative modeling approach that models individual differences while not requiring dysfunctions or alterations to overlap (Marquand et al., 2019). Notably, this method has already been successfully applied to model development in schizophrenia, bipolar disorder (Wolfers et al., 2018) and autism (Bethlehem et al., 2020; Floris et al., 2021; Zabihi et al., 2019). Given previous evidence for abnormalities in emotion processing and its underlying structures, we first applied normative modeling (Marquand et al., 2019) to a cross-sectional dataset, where variation in each time point across individuals is sampled, to investigate how DBD cases deviate from an expected pattern of brain development. Second, since DBD is likely to be explained by multimodal neurobiological variation, we integrated structural and functional deviations from two different imaging modalities by linked independent component analysis [LICA (Groves, Beckmann, Smith, & Woolrich, 2011; Llera, Wolfers, Mulders, & Beckmann, 2019)] to provide a comprehensive model to explain aggressive behavior at a symptom level.

## Method

### Study sample

Participants in the current study were part of the EU-funded Aggressotype and MATRICS projects. The assessment followed a strict standard operating protocol, which was consistent across all sites. The first day consisted of a standardized interview, questionnaires to assess psychopathology, behavior, and sociodemographic information and a neuropsychological assessment (approximately 3 h). On the second day, the participants took part in an MRI session (approximately 1.5 h). In total 208 participants (129 cases, 79 controls) aged 8–18 years were assessed using functional MRI (fMRI) and anatomical scans were acquired in *n* = 248 (154 cases, 94 controls) across nine sites in Europe. Exclusion criteria for all participants were any contraindications for MRI, an IQ<80 [as measured by four subtests (vocabulary, similarities, block design, and picture completion/matrix reasoning) of the Wechsler Intelligence Scale for Children-IV (Wechsler, 2011)], and a primary DSM-5 diagnosis of psychosis, bipolar disorder, major depression, and/or an anxiety disorder. Participants who were included as cases with DBD were diagnosed with ODD and/or CD based on the structured diagnostic interviews with the child and parents according to DSM-5, and/or scored above the clinical cut-off for aggressive behavior and/or rule-breaking behavior as measured with the Child Behavior Checklist completed by parents, teachers and/or youths themselves [CBCL/TRF/YSR; (Achenbach, Howell, Quay, & Conners, 1991)]. In the control group, no DSM axis I disorder, assessed via the K-SADS (Kaufman et al., 1997), and no clinical score in the CBCL, TRF or YSR was allowed, according to standard cutoffs for these instruments. For cases with DBD, medication had to be stable for at least two weeks prior to inclusion. The parent-rated Inventory of Callous-Unemotional Traits (ICU) (Essau, Sasagawa, & Frick, 2006) and the self-reported Reactive Proactive Aggression Questionnaire (RPQ) (Raine et al., 2006) were used to assess features of aggression. ADHD symptoms were measured with the parent-rated SNAP-IV questionnaire (Bussing et al., 2008). Pubertal status was assessed with the Pubertal Development Scale (Petersen, Crockett, Richards, & Boxer, 1988). The internal consistency of all measures ranged from good to excellent (for details see Rosa-Justicia et al., 2022). Correlations between all aggression measures are provided in the online Supplementary Fig. S1. The participants received 85 € for their participation. Ethical approval for the study was obtained for all sites separately by local ethics committees. The authors assert that all procedures contributing to this work comply with the ethical standards of the relevant national and institutional committees on human experimentation and with the Helsinki Declaration of 1975, as revised in 2008. Written informed consent was given by the participants and their parents or legal representatives.

### fMRI task

Participants performed an emotional face-matching task (Hariri, Bookheimer, & Mazziotta, 2000). In total two emotion matching blocks and two blocks of a sensorimotor control task were presented. During the first, one block consisted of six stimuli depicting a trio of faces with either negative (anger and fear) or positive/neutral faces (happy and neutral) in which the participants had to select one of two emotions (displayed on the bottom) identical to the target stimulus (displayed on the top). Interleaved between these blocks, participants completed two blocks of a sensorimotor control task with one block consisting of six geometric shapes (horizontal ellipses or vertical ellipses) that had to be matched accordingly (for details see online Supplementary Fig. S2 in the Supplementary material). Subjects responded by pressing one of two buttons with their right hand according to the match in emotion or shape. The task lasted 3:57 min. This task robustly elicited the face-processing network in this sample, including the amygdala (Aggensteiner et al., 2020).

### Functional images

MRI scans were performed in nine different sites across Europe. Whole-brain functional emotion matching data were acquired with echo-planar T2*-weighted imaging (EPI), sensitive to the Blood Oxygenation Level Dependent (BOLD) signal contrast (36 axial slices, 3 × 3 × 3 mm, slice gap 0.4 mm, TR = 2100 ms, TE = 35 ms, see Supplementary material text and online Supplementary Table S1). Data were analyzed using SPM12 (https://www.fil.ion.ucl.ac.uk/spm/) with standard preprocessing steps. Participants with head movement >3 mm or 3° were excluded. Data from five sites were included in the final analysis to reach a minimum of five cases and controls per site, resulting in a sample of *N* = 140 (85 cases). A further 11 participants were excluded due to residual movement artefacts in the GLM regression coefficients and the *Z* statistic images [*N* = 129 (77 cases), see supplement]. There was no systematic bias with regard to demographic and clinical data between included and excluded data sets (see Supplementary material and online Supplementary Table S2). To harmonize the data regarding site effects, scanner difference was modeled and removed from the scans using ComBat (Johnson, Li, & Rabinovic, 2007).

### Anatomical images

In addition to the functional data, an additional high-resolution structural magnetization-prepared rapid gradient echo (MP-RAGE) scan was also acquired at a resolution of 1 × 1 × 1.2 mm. Scan parameters derived from the ADNI-2/ADNI-Go project (Jack et al., 2010) were consistent across the four sites using a Siemens scanner (TR = 2300, TE = 2.96/2.98) and comparable at the GE site, see online Supplementary Table S3. The complete anatomical data set comprised 248 participants. Out of this sample, *n* = 129 had both functional and structural data available. Two scans had to be excluded due to bad image quality, leaving a final sample of 127 participants. Pre-processing was done using the anatomical processing tools implemented in FSL (details described in the Supplementary material). For further analyses, Jacobian determinants (JDs) of the deformation fields were used as anatomical features (see Supplementary material). In a further step, scanner variance in these images was removed using ComBat (Johnson et al., 2007).

### Constructing normative models of brain structure and function

**Fig. 1.**
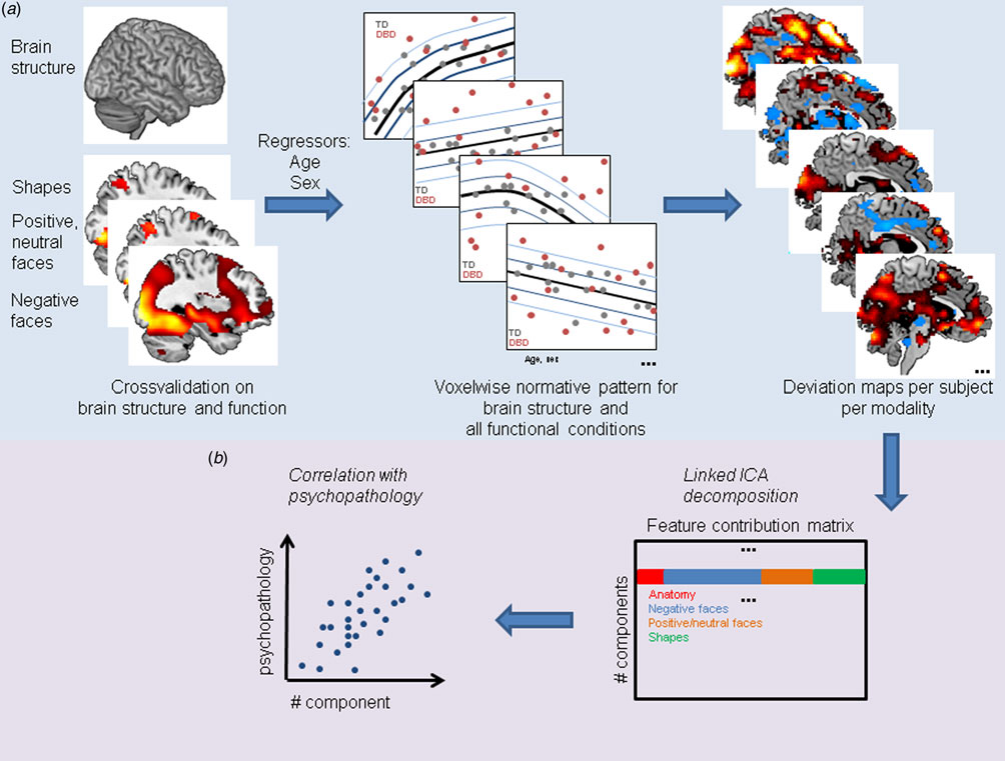
Methodological overview. (*a*). A normative model was estimated from brain structure and function across typically developing controls (TDs) and DBD cases, which allowed us to estimate normative probability maps, showing the regional deviations from the expected pattern in each subject. (*b*) These deviations were integrated using linked ICA, which yielded 20 components that were related to behavioral aggression measures.

A model of brain development as a function of age and sex was created by training a Gaussian process regression (GPR) model, which is a nonparametric nonlinear regression technique with additive Gaussian noise, on a sample of *n* = 129 and *n* = 127 (77 cases, Table 1, online Supplementary Fig. S3 for an age distribution) to predict brain function and structure, respectively (Fig. 1*a*). The model can be set up in two ways, using either the whole cohort (both healthy participants and cases) or only the healthy participants as the reference cohort. Applying either has been shown to make little difference (Marquand et al., 2019). Here, we used the whole cohort (both TD and DBD) as a reference due to the small sample size of the intersection between anatomical and functional data implying that the frequency of DBD in the normative model (*n* = 77) is higher than in the control population, which means that results ought to be interpreted with respect to this specific cohort. Note that in our analysis GPR predictions are derived in an unbiased manner under 10-fold cross-validation. Briefly, this Bayesian approach calculates the probability distribution over all functions that fit the data while specifying prior over all possible values and relocating probabilities based on evidence (i.e. observed data). As such, it yields unbiased estimates of generalizability and inferences with increasing uncertainty with fewer data. This, in turn, increases the conservativeness of this approach and renders deviations harder to detect.

**Table 1.** Sample characteristics

	Control (*n* = 52)	Case (*n* = 77)	Test statistics
Males, no. (%)	34 (65.4)	64 (83.1)	χ^2^ = 5.35, *p* = 0.02
Age, mean (s.d.)	13.88 (2.44)	13.48 (2.58)	*T*(127) = 0.88, *p* = 0.38
IQ, mean (s.d.)	108.55 (10.95)	99.18 (10.13)	*T*(127) = 4.99, *p* < 0.001
ADHD diagnoses, no. (%)	0	20 (25.97)	χ^2^ = 17.22, *p* < 0.001
CBCL externalizing problems, mean (s.d.)	4.45 (4.86)	29.03 (11.39)	*Z* = −9.023, *p* < 0.001
CBCL conduct problems, mean (s.d.)	1.33 (2.06)	11.53 (5.90)	*Z* *=* −8.82, *p* < 0.001
CBCL oppositional defiant, mean (s.d.)	1.67 (1.84)	7.24 (2.13)	*Z* = −8.94, *p* *<* *0.*001
CBCL rule breaking, mean (s.d.)	1.37 (2.00)	9.68 (6.25)	*Z* = −8.18, *p* < 0.001
CBCL aggression, mean (s.d.)	3.10 (3.21)	19.52 (7.21)	*Z* = −9.13, *p* < 0.001
RPQ proactive, mean (s.d.)	0.98 (1.58)	4.33 (4.05)	*Z* = −5.46, *p* < 0.001
RPQ reactive, mean (s.d.)	5.90 (3.61)	12.17 (4.45)	*Z* = −6.73, *p* < 0.001
RPQ total, mean (s.d.)	6.88 (4.65)	16.50 (7.29)	*Z* = −6.86, *p* < 0.001
ICU total, mean (s.d.)	20.04 (8.05)	32.05 (10.10)	*Z* = −6.18, *p* < 0.001
ICU callousness, mean (s.d.)	3.88 (2.25)	8.36 (4.81)	*Z* = −5.67, *p* < 0.001
ICU uncaring, mean (s.d.)	9.58 (5.14)	13.96 (5.22)	*Z* = −4.40, *p* < 0.001
ICU unemotional, mean (s.d.)	5.40 (2.63)	7.60 (3.43)	*Z* = −3.70, *p* < 0.001
ADHD symptoms, mean (s.d.)	6.02 (7.2)	31.15 (12.53)	*Z* = −8.43, *p* < 0.001
Medication (%)	0	51 (66.23)	χ^2^ = 56.96, *p* < 0.001
Stimulants		39%	
Antipsychotics		20%	
Antidepressants		2.6%	
Other		2.6%	

As a sensitivity analysis, we leveraged our larger anatomical dataset (*n* = 248) before the intersection with the smaller fMRI data set to compare results using different reference cohorts, which yielded similar results (see online Supplementary Table S4).

To estimate a pattern of regional deviations from typical brain structure and function for each participant, we derived a normative probability map (NPM) that quantifies the voxel-wise deviation from the normative model. This was done by using the normative model to predict estimates of brain structure and function for each individual participant, then calculate a subject-specific *Z* score (Marquand, Rezek, Buitelaar, & Beckmann, 2016) indicating the difference between the predicted and true brain activity (or structural measure) scaled by the prediction variance. The NPMs were thresholded at *Z* = ± 2.6 (i.e. *p* < 0.005) as in Floris et al. (2021), Wolfers et al. (2018) to facilitate the comparison across participants and to have a more sensitive marker for small individual deviations when compared to false discovery rate correction.

The total number of (absolute) mean deviations from DBD cases and controls were extracted and compared using the non-parametric Mann–Whitney *U* Test. Multiple comparison were controlled for using FDR correction with the Benjamini–Hochberg method (Benjamini & Hochberg, 1995).

Mean threshold-free, cluster-enhanced group differences between DBD cases and controls were estimated using Permutation Analysis of Linear Models (PALM) (Winkler, Webster, Vidaurre, Nichols, & Smith, 2015) on the normative deviation maps and family-wise error corrected for multiple comparisons.

### Linked ICA

We used linked ICA (Groves et al., 2011; Llera et al., 2019) to integrate the unthresholded normative (Z-transformed) deviation maps across four data modalities: negative faces, positive/neutral faces, and shapes as well as anatomy (Fig. 1*b*) to tackle the potential overlap of effects across brain regions. Each measure is considered as a different ‘modality’ in this context in the sense that the algorithm will learn the different noise characteristics for each of these data types. Linked ICA is a Bayesian multimodal extension of the ICA model (Groves et al., 2011; Llera et al., 2019) that provides simultaneous factorizations across multiple data modalities, while linking them at the subject level through a shared mixing matrix reflecting subject-wise contributions to each independent component (1 scalar value per participant). Further, each independent component provides also a map of spatial variation per modality and a vector reflecting the relative contribution of each modality to the component (Beckmann & Smith, 2005). Given our sample size and following recommendations described in earlier papers (Wolfers et al., 2017), linked ICA was used to estimate 20 independent components. Although the results were not critically dependent on this parameter value, such model order provided also a superior performance in predicting DBD cases from controls when compared to 30 independent components in terms of Akaike Information Criterion (AIC) and the Bayesian Information Criterion (see online Supplementary Fig. S4). For visualization, the spatial maps were converted to pseudo-*Z*-statistics and thresholded at *|Z|* > 2.6.

### Behavioral correlatess

Given the high correlations between the aggression measures (online Supplementary Fig. S1), a principal component analysis with VARIMAX rotation was performed that included the DSM-oriented subscales conduct problems and oppositional defiant problems from the CBCL as well as the total ICU score and reactive and proactive aggression from the RPQ. This yielded one factor that explained 67% of the variance in the aggression measures. Spearman correlations were performed to relate the deviations from each modality separately to the aggression factor. Further, to assess how combined deviations across all modalities act in concert to explain psychopathology, all 20 components were entered into a logistic regression to predict DBD diagnosis. Further, each of the 20 linked ICA components was associated with the aggression factor using FDR correction and was tested as being different between cases and controls using Mann–Whitney *U* test.

### Sensitivity analyses

Significant correlations between relevant independent components and the aggression factor were further controlled for IQ, binary medication use, internalizing symptoms, ADHD symptoms and pubertal status using nonparametric partial correlations.

In addition, we fed the unmodeled contrast maps and JD's (i.e. without fitting any normative model) in the LICA, which indicated a better model fit of the linked ICA based on the deviation scores to predict DBD (details depicted in the supplement). While this was not an explicit aim of this study, our findings illustrate a benefit for normative modeling relative to applying stratification tools to the data directly. This can be attributed to the accurate, non-linear modeling of demographic characteristics using GPR and the placement of each subject with respect to centiles of variance in a common reference model. Importantly, this yields *z*-statistics that are consistently calibrated across modalities which enables the subsequent LICA to explain slightly more variance and – more importantly – detect stronger associations with categorical and dimensional clinical measures. This is in line with recent evidence of the increased predictive performance of cortical volume deviation scores over raw scores regarding psychopathology (Parkes et al., 2021).

## Results

### Sample

Out of the 77 included cases, 33 were diagnosed with ODD, four with CD, and 15 with ODD/CD. The other 25 participants were included as ‘clinical case’ based on a CBCL/TRF/YSR aggression and/or rule-breaking behavior subscale *T* ⩾ 70. As expected, the case group consisted of more males, had a lower IQ, and scored higher on all measures of aggression (see online Supplementary Fig. S5 for the distribution) and ADHD as compared to controls (Table 1). No differences across site or site by group effects were seen regarding demographics and clinical variables (online Supplementary Table S5). Cases and controls did not differ regarding the movement in the scanner (translation: *U* = 1755, *Z* = −1.186, *p* = .24; rotation: *U* = 1663, *Z* = −1.63, *p* = 0.11) or task performance.

### Normative models

The accuracy of the normative models for predicting brain structure and function was evaluated using the correlation between the true and the predicted voxel values *ρ* for cases and controls (online Supplementary Fig. S6).

**Fig. 2.**
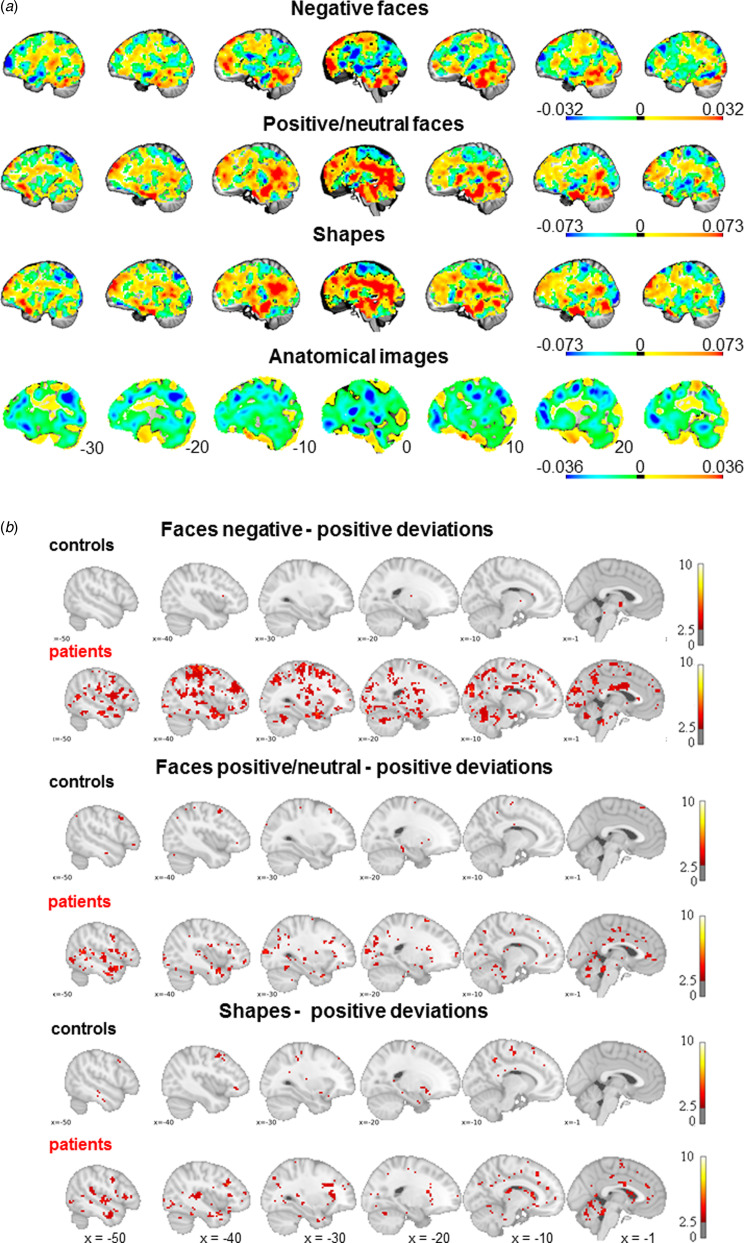
(*a*) Spatial representation of the voxel-wise normative model for all modalities in males. The panels show the beta values depicting the change across 8 to 18 years of age for negative face processing, positive/neutral face processing, shape processing and anatomical images. Red and blue indicate an increase or decline, respectively, in activity or relative expansion or contraction to match the template for the Jacobian determinants. (*b*) Mean positive deviations from the model for controls and cases. Cases showed significantly more positive deviations, i.e. a higher age-related increase in prefrontal and limbic (i.e. cluster comprising the amygdala) activity presumably during negative and positive/neutral face matching. For visualization purposes only the left side is shown.

As shown in Fig. 2*a*, decreases as well as increases of brain activity are observed in the functional images, while mostly relative contractions are seen in the anatomical images (see Supplementary material for details).

### Normative modeling-mean age-related deviations

**Fig. 3.**
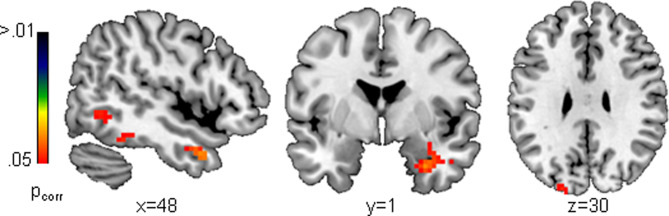
Cases showed increased deviations, i.e. higher age-related activity, from the normative model during negative face processing in the amygdala, the parahippocampal gyrus, the (inferior) temporal gyrus, the fusiform gyrus and the cuneus when compared to controls. This is suggestive of delayed maturational trajectories of brain activation in DBD cases.

PALM analyses yielded significant group differences in regional deviations including the parahippocampal gyrus/amygdala, the fusiform gyrus, the middle temporal gyrus and the cuneus for negative faces only (Fig. 3). Thus, this indicates that – on average – cases exhibit more age-related activation deviations in these regions, which can be considered as an age-by-diagnosis interaction.

### Normative modeling-DBD cases show more positive deviations

Participants with DBD showed more individual positive deviations when compared to controls with regard to all functional measures (Fig. 2*b*; online Supplementary Table S6 in the Supplementary material) indicating relative age-related increases in activity from childhood to late adolescence at the individual level (that are not necessarily consistent across the cohort). No significant differences emerged with regard to negative deviations and with regard to the JDs (all comparisons *p* > 0.05).

#### Behavioral associations

While the correlation between positive deviations from the normative model of negative face processing and the aggression factor fell short of significance after FDR correction (*ρ* = .19, *p*_FDR_ = 0.06), deviations from the normative model of positive/neutral face processing were significantly associated with aggression (*ρ* = 0.24, *p*_FDR_ = 0.036). No significant correlations were found regarding deviation from shape processing.

### Linked ICA

**Fig. 4.**
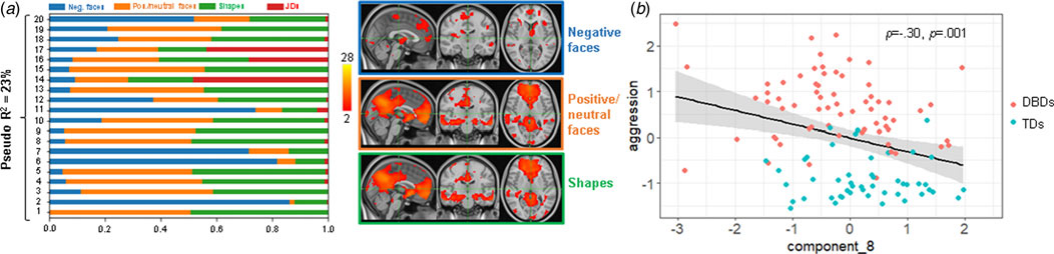
Linked ICA. (*a*). Loading matrix showing the contribution of each modality to each of the 20 components (blue: negative faces, orange: positive/neutral faces, green: shapes, red: anatomy). The underlying brain pattern of component 8 (in the matrix number 8) per functional modality is depicted on the right side (thresholded at *Z* > 2). (*b*) Component 8 was negatively associated with the aggression factor, which was still significant when outliers were excluded (*p* = 0.003).

Each of the 20 linked ICA components represents the spatial pattern of the deviations. The components are depicted in Fig. 4*a*, i.e. the loadings of each modality for each component. Participants' loadings of each of the 20 imaging markers based on the deviation maps of the functional and anatomical data together predicted 23% (Mc Faddens *R*^2^) of the variance of DBD (χ^2^ = 39.2, *df* = 20, *p* = 0.006; Fig. 4*a*). Two components significantly differed between cases and controls at the nominal level (component 5: *U* = 1515, *Z* = −2.08, *p*_uncorr_ = 0.04, *p*_FDR_ = 0.28, component 8: *U* = 1417, *Z* = −2.56, *p*_uncorr_ = 0.01, *p*_FDR_ = 0.20) but did not survive FDR correction. The latter multimodal component (number 8) showed additional associations with the aggression factor across the whole sample that survived multiple comparison correction (*p*_FDR_ = 0.02). This component loaded most strongly on deviations from positive/neutral faces and shape processing with a negligible loading on anatomic deviations (Fig. 4*b*) and explained 9% of the variance in the aggression factor. The corresponding spatial maps (Fig. 4*a* right side) show that component 8 is strongly reflective of the default mode network (DMN), with high weightings in the precuneus and the medial prefrontal cortex. Moreover, both positive and neutral face and shape processing show additional weightings in the (ventral) striatum (nucleus accumbens, caudate), the amygdala/hippocampus, and the temporal gyrus, while the spatial map of negative face processing loaded also to the thalamus, caudate, post- and precentral gyrus, and superior frontal gyrus. The results remained unchanged or similar after the sensitivity analyses (presented in the Supplementary material).

## Discussion

The present investigation provides several novel findings in relation to DBD: First, we provide evidence for aberrant age-related brain structure and function in cases with DBD. As such, on average, DBD cases showed increased deviations from the normative pattern in the face-processing network, including the fusiform gyrus, the amygdala, and the temporal gyrus during negative affective processing, which is indicative of higher age-related activity when compared to controls. Second, by moving beyond case–control differences, our normative modeling approach showed striking, widespread patterns of more positive age-related deviations in and beyond the face processing circuit in the DBD cases that are indicative of an increased age-related activity at the level of the individual. Third, assuming multi-faceted manifestations across multiple quantitative phenotypes in aggression, the integration of modalities explained almost one fourth of the variance in DBD symptomatology, thus revealing that the inter-individual variation across these phenotypes is matched. The most predictive marker for aggression on a continuous level was multimodal, predominantly affected by positive/neutral face processing and shape processing. Taken together, our results (i) provide the first evidence for neurodevelopmental atypicalities reflected in higher age-related brain activity in DBD, (ii) underscore the importance of moving beyond case–control comparisons and provide inferences at the level of the individual and (iii) show the advantage to integrate across multiple data modalities.

Our normative modeling approach suggests that cases with DBD have a higher age-related activity during face processing that was region-specific at the group-level and widespread at the level of the individual. The latter is reflected in widespread non-focal patterns of atypical activation which might have been overshadowed by classical case–control differences that per nature require a consistent pattern of neurobiological variation within groups. Interestingly, only positive deviations were observed and classical case–control analyses emphasized age-related deviations in the amygdala during negative face processing. Specifically, in the right amygdala the normative model predicted a gradual decrease in activity as a function of age during negative face processing. Thus, one may posit that positive deviations in this region as seen in DBD, which imply a higher activity with respect to the trajectory as expected in the current sample, could point to a maturational delay. Note, however, that this interpretation might not be valid for other brain regions which followed different age trajectories as shown in Fig. 2*a*. Further, due to the cross-sectional nature of our study this interpretation ought to be further validated in large longitudinal studies enabling to make inferences about neurodevelopmental trajectories of the face-processing network across several modalities. Overall, this can complement the picture of a less synchronized pattern of brain development in adolescent-onset CD (Fairchild et al., 2016) and altered developmental trajectories of emotion-relevant brain structures in DBD (Hummer, Wang, Kronenberger, Dunn, & Mathews, 2015). In addition to higher activity during face processing, age-related positive deviations in shape processing were observed. This may point to abnormal cognitive perceptual processing, which might be attributable to increased ADHD symptoms in cases (Fuermaier et al., 2018).

The combination of several markers of deviation showed robust associations with DBD caseness. The top component related to aggression on a symptom level showed focal effects, presumably in the precuneus and the medial prefrontal cortex, which strongly resemble the DMN. Notably, this is in line with reports of reduced functional connectivity of the DMN in DBD (Broulidakis et al., 2016) and positive associations between abnormalities of the DMN and impulsivity scores (Lu, Zhou, Zhang, Wang, & Yuan, 2017). Given the role of the DMN in self-referential cognitive processing (Andrews-Hanna, Reidler, Sepulcre, Poulin, & Buckner, 2010), age-related deviations in the DMN might further contribute to deficient emotion and empathy processing that is often observed in DBD (Blair et al., 2015; Fairchild et al., 2019).

Interestingly, the ventral striatum and the amygdala also loaded onto the top ICA component, which echoes previous reports emphasizing the importance of these structures in DBD (Fairchild et al., 2019). The amygdala has been suggested to be one of the core regions disrupted in structure and function in DBD, thereby, likely contributing to the observed deficits in emotion recognition and affective empathy (Blair et al., 2018; Fairchild et al., 2019). Notably, the striatum, including the caudate, has been highlighted as being dysfunctional during reward processing with blunted activity during anticipation (Holz et al., 2017), impaired representation of expected value information and increased punishment-related prediction error representation (White et al., 2013). In sum, our findings are suggestive of a dysfunctional affective circuit, and provide the first evidence for multimodal age-dependent variation that are related to aggression, which, in turn, might result in impulsive decisions and frustration (Blair et al., 2018).

While this study employed a well-phenotyped sample of cases with DBD and healthy controls from a harmonized multicenter cohort, several caveats should be taken into account. First, even larger sample sizes would allow for improved normative modeling, e.g. permitting to derive normative models for TDs only. However, given the similar results in the larger anatomical data set with TDs only as reference cohort, it can be assumed that the normative model across the whole sample is not biased (Marquand et al., 2019). Second, while our data allowed investigating aggression dimensions, the sample size did not allow for a distinction between early onset and adolescent-onset DBD, the latter being linked to a less severe developmental trajectory (Fairchild et al., 2019). However, evidence suggests that both subgroups exhibit deficits in core regions of emotion processing, such as the amygdala (Passamonti et al., 2010). Third, our normative model was constructed based on age and sex. While the findings are suggestive of sex differences, our sample size does not allow to statistically test this. Future studies with more power should investigate this interesting topic. Fourth, given that our results are based on a cross-sectional data set, future longitudinal studies are warranted to further examine individualized brain development in DBD. Fifth, the nature of the task which included simultaneous matching of neutral and positive faces might have diluted subtle effects. Based on our results of increased age-related differences at the individual level during this condition, future studies should disentangle whether individual neurodevelopmental deviations in DBD exist specifically for positive face processing. Sixth, the inclusion of participants with clinical aggression scores in the CBCL renders the DBD group more heterogeneous, which is a limitation in the case–control comparisons at the behavioral level but supports the use of the individual-based normative modeling approach.

In conclusion, our findings add substantially to our understanding of DBD. On the one hand, we used normative modeling to map the heterogeneous biological variation in brain function and structure underlying DBD as a function of age. We provide clear evidence for age-related differences in the affective circuit with involvement of the amygdala for these disorders and make progress toward developing individualized inferences in the spirit of precision medicine. On the other hand, our integrated analysis using linked ICA identifies the DMN along with the amygdala and the striatum, as common disrupted neurobiological signatures that may contribute to the emergence of aggression.

## Supplementary material

For supplementary material accompanying this paper visit https://doi.org/10.1017/S003329172200068X. click here to view supplementary material
