# Supplementary material for: Age-related brain deviations and aggression
Source: Psychol Med. 2022 Apr 22;53(9):4012–21. doi: 10.1017/S003329172200068X (PMC10325848; doi:10.1017/S003329172200068X)
Supplement: Supplementary file 1 [file S003329172200068Xsup.zip › S003329172200068Xsup007.docx]

**Integrating age-related functional and structural brain deviations to characterize disruptive behavior disorders**

**Running Title: Age-related brain deviations and aggression**

Nathalie E. Holz*^1^, PhD, Dorothea L. Floris*^2,3^, PhD, Alberto Llera*^2^, PhD, Pascal M. Aggensteiner^1^, PhD, Seyed Mostafa Kia^2,3^, PhD, Thomas Wolfers^2,3,4^, PhD, Sarah Baumeister^1^, PhD, Boris Böttinger^1^, PhD, Jeffrey C Glennon^3^, PhD, Pieter J Hoekstra^5^, PhD, Andrea Dietrich^5^, PhD, Melanie C Saam^6^, Msc, Ulrike M E Schulze^6^, MD, David J Lythgoe^7^, PhD, Steve C R Williams^7^, PhD, Paramala Santosh^8,9^, MD, PhD, Mireia Rosa-Justicia^10,11^, PhD, Nuria Bargallo^10^, MD, PhD, Josefina Castro-Fornieles^12^, MD, PhD, Celso Arango^13^, MD, PhD, Maria J Penzol^13^, MD, PhD, Susanne Walitza^14^, MD, Andreas Meyer-Lindenberg^15^, MD, PhD, Marcel Zwiers^2^, PhD, Barbara Franke^16,17^, PhD, Jan Buitelaar^2,3,18^, MD, PhD, Jilly Naaijen^2,3^, PhD, Daniel Brandeis^1,12,19,20^, PhD, Christian Beckmann†^2,3,21^, PhD, Tobias Banaschewski†^1^, MD, PhD, Andre F. Marquand†^2,3,7,^ PhD

^1^Department of Child and Adolescent Psychiatry and Psychotherapy, Central Institute of Mental Health, Medical Faculty Mannheim/Heidelberg University, Mannheim, Germany

^2^Donders Center for Brain, Cognition and Behavior, Radboud University Nijmegen, Nijmegen, the Netherlands

^3^Department for Cognitive Neuroscience, Radboud University Medical Center Nijmegen, Nijmegen, the Netherlands

^4^NORMENT, KG Jebsen Centre for Psychosis Research, Division of Mental Health and Addiction, Oslo University Hospital & Institute of Clinical Medicine, University of Oslo, Oslo, Norway

^5^University of Groningen, University Medical Center Groningen, Department of Child and Adolescent Psychiatry, Groningen, The Netherlands

^6^Department of Child and Adolescent Psychiatry/Psychotherapy, University Hospital, University of Ulm, Germany

^7^Department of Neuroimaging, Institute of Psychiatry, Psychology & Neuroscience, King`s College London, London, UK

^8^Department of Child Psychiatry, Institute of Psychiatry, Psychology & Neuroscience, King`s College London, London, UK

^9^Centre for Interventional Paediatric Psychopharmacology and Rare Diseases (CIPPRD), South London and Maudsley NHS Trust, London, UK

^10^Clinic Image Diagnostic Center (CDIC), Hospital Clinic of Barcelona; Magnetic Resonance Image Core Facility, IDIBAPS, Barcelona, Spain

^11^Child and Adolescent Psychiatry and Psychology Department, Institute Clinic of Neurosciences, Hospital Clinic of Barcelona, IDIBAPS, Spain

^12^Child and Adolescent Psychiatry and Psychology Department, 2017SGR881, Institute Clinic of Neurosciences, Hospital Clinic of Barcelona, CIBERSAM, IDIBAPS, Department of Medicine, University of Barcelona, Barcelona, Spain

^13^Child and Adolescent Psychiatry Department, Institute of Psychiatry and Mental health, Hospital General Universitario Gregorio Marañón School of Medicine, Universidad Complutense, IiSGM, CIBERSAM, Madrid, Spain

^14^Department of Child and Adolescent Psychiatry and Psychotherapy, Psychiatric Hospital, University of Zurich, Zurich, Switzerland

^15^Department of Psychiatry and Psychotherapy, Central Institute of Mental Health, Medical Faculty Mannheim/Heidelberg University, Mannheim, Germany

^16^Radboud University Medical Center, Department of Human Genetics, Nijmegen, The Netherlands

^17^Radboud University Medical Center, Department of Psychiatry, Nijmegen, The Netherlands

^18^Karakter Child and Adolescent Psychiatry University Center, Nijmegen, The Netherlands

^19^Center for Integrative Human Physiology, University of Zurich, Zurich, Switzerland.

^20^Neuroscience Center Zurich, University of Zurich and ETH Zurich, Zurich, Switzerland.

^21^Centre for Functional MRI of the Brain, University of Oxford, Oxford, United Kingdom

*shared first authorship

† shared last authorship

Correspondence to:

Nathalie Holz, Department of Child and Adolescent Psychiatry and Psychotherapy, Central Institute of Mental Health, Medical Faculty Mannheim / Heidelberg University, J 5, 68159 Mannheim, Germany, Tel: +49 621/1703-4904, E-mail: nathalie.holz@zi-mannheim.de

Andre F. Marquand, Donders Centre for Cognitive Neuroimaging, Donders Institute for Brain, Cognition and Behaviour, Radboud University, Kapittelweg 29, 6525 EN Nijmegen, The Netherlands, Tel: +31 (0)24 366 84 92, E-mail: a.marquand@donders.ru.nl

**Supplement**

# Methods

## Functional images

The first five volumes were discarded to allow longitudinal magnetization to reach equilibrium. EPIs were interpolated in time to correct for slice time differences and realigned to the first scan to correct for head movements. EPIs were co-registered and normalized to standard EPI template implemented in SPM using linear and non-linear transformations and smoothed with a full-width-half-maximum Gaussian kernel of 8 mm. Three vectors comprising onsets and durations of either shapes or negative or positive/neutral faces were convolved with the SPM8 canonical hemodynamic response function in the context of a General Linear Model in order to model the BOLD time course. A further six movement parameters were included as regressors of no interest.

The GLM regression coefficient images and the Z images (deviation images) of the normative model of the 140 participants were visually inspected by an experienced rater (NH) to identify residual movement artefacts, which resulted in an exclusion of 11 participants, yielding a final sample of n=129 (77 cases). To achieve a more precise normative model, the whole cross-validation was rerun in this sample, which was used for further analyses.

To guarantee no systematic sample bias by these strict exclusion criteria, we ensured that there was no difference between our sample when compared to the excluded sample (n= 48, Table S3) that was included in the case-control analysis as described by Aggensteiner et al. (2020).

## Anatomical images

Preprocessing of the anatomical images entailed the following steps. First, images were reoriented to the standard (MNI) orientation [fslreorient2std], automatically cropped [robustfov] and bias-field corrected (RF/B1-inhomogeneity-correction) [FAST]. Then registered to standard space (linear and non-linear) [FLIRT and FNIRT], followed by brain-extraction [FNIRT-based or BET] as well as tissue-type segmentation [FAST] and subcortical structure segmentation [FIRST]. Data were visually inspected and evaluated by experienced raters using a rating system that has been described and thoroughly applied to an MRI data-set of children with ADHD and/or CD before (Backhausen et al., 2016).

We decided to take the nonlinear Jacobian determinants as feature for the normative model given that they quantify the overall degree of tissue expansion or contraction required to match each image to the population template. Thereby, they contain all information necessary to warp the subject to template and are no arbitrary distinction between white and grey matter.

# Results

## Normative models

As shown in Figure 2A, the voxel-wise normative model depicting the processing of negative faces predicted an increase in brain activity from 8 to 18 years of age, particularly in the superior frontal gyrus, the lingual gyrus, the brain stem, the occipital gyrus and cerebellar regions, the medial prefrontal cortex, the parahippocampal gyrus, including the amygdala for men. A decrease in activity from childhood to late adolescence can be observed in the cingulate gyrus, the frontal and temporal pole, the inferior frontal gryus, the middle frontal gyrus, the superior temporal gyrus, the thalamus and the amygdala.

Stronger and more similar increases in activity with age can be seen during processing of positive/neutral faces and shapes. Specifically, increases can be observed in the temporal pole, the inferior, middle and superior frontal gyrus, the left orbital gyrus, the brain stem, the (anterior) cingulate gyrus, the precuneus, the amygdala (faces only), the hippocampus and decreases more in the insula, the amygdala, the superior temporal gyrus, the inferior frontal gyrus and the supplementary motor cortex (shapes only).

In contrast, the normative model of brain structure was characterized by a relative contraction of deformation fields such as in the supramarginal gyrus, the middle temporal, frontal and occipital gyrus, cingulate gyrus, postcentral gyrus, frontal medial cortex and the insula, except for the middle frontal gyrus, and the superior parietal lobule, where a relative expansion is observed.

## Sensitivity analyses of the Linked ICA analyses

Correlations between component 8 and the aggression factor remained significant when controlled for IQ (*ρ*(110)= -.30, *p*=.001), medication (*ρ* (110)= -.23, *p*=.01), pubertal status (*ρ* (105)= -.28, *p*=.003) and got even stronger when internalizing symptoms were added as a covariate (*ρ*(109)= -.31, *p*=.001). When controlling for ADHD the correlation fell short of significance (*ρ* (100)= -.19, *p*=.05).

Feeding the contrast images and JD´s (i.e. without fitting any normative model) in the LICA also yielded a significant association with DBD caseness when corrected for age and sex (X2=35.25, df=22, p=.07, AIC=182.6, BIC=248.0, Mc Faddens R2=20%), although it should be emphasized that this correction was performed in-sample whereas the normative model always estimated Z-statistics using out of sample prediction (i.e. on unseen data). However, a slightly increased model fit is found when predicting DBD based on the components reflecting the deviation scores (X2=39.2, df=20, p=.006, AIC=173.9, BIC=233.6, Mc Faddens R2=23%). This model superiority is further confirmed by the lack of associations between the components of the raw data with aggression (all p>.09).

# References

Aggensteiner, P. M., Holz, N. E., Bottinger, B. W., Baumeister, S., Hohmann, S., Werhahn, J. E., . . . Brandeis, D. (2020). The effects of callous-unemotional traits and aggression subtypes on amygdala activity in response to negative faces. *Psychological Medicine*, 1-9. doi:10.1017/S0033291720002111

Backhausen, L. L., Herting, M. M., Buse, J., Roessner, V., Smolka, M. N., & Vetter, N. C. (2016). Quality Control of Structural MRI Images Applied Using FreeSurfer-A Hands-On Workflow to Rate Motion Artifacts. *Frontiers in Neuroscience, 10*, 558. doi:10.3389/fnins.2016.00558

# Figure Legends

Figure S1. Correlation matrix. *** p<.001, ** p<.01, * p<.05

Figure S2. Task design.

Figure S3. Age distribution.

Figure S4. The Akaike Information Criterion (AIC) and the Bayesian Information Criterion (BIC) for the logistic regression predicting DBD casesness with 30 components and 20 components.

Figure S5. Violin plots depicting the distribution of the aggression measures across cases and controls.

Figure S6. The figure shows the correlation (Rho) between true and predicted brain activity and deformation fields..

| Table S1. Functional MRI scan parameters across sites. | | | | | |
| --- | --- | --- | --- | --- | --- |
| **Scanner** | **Site** | **TR/TE (ms)** | **Number of slices** | **Slice scan order** | **Voxel size (mm)** |
| Siemens | Nijmegen | 2100/35 | 36 | descending | 3x3x3 |
|  | Mannheim | 2100/35 | 36 | descending | 3x3x3 |
|  | Barcelona | 2100/35 | 36 | descending | 3x3x3 |
|  | Madrid | 2100/35 | 36 | descending | 3x3x3 |
| GE* | London | 2100/35 | 36 | descending, sequential | 3x3x3 |
| *All sites used a 32-channel head coil except for the general Electric 3-Tesla scanner (8-channel head coil). The GE site also used an Inversion Recovery Spoiled Gradient Recalled Echo (IR-SPGR) | | | | | |

Table S2. Attrition analysis.

|  | Excluded Sample (N=48) | Included sample (N=129) | Statistics |
| --- | --- | --- | --- |
| Cases, No. | 31 | 77 | Χ^2^=.35, p=.61 |
| Age, mean (SD) | 13.03 (3.02) | 13.64 (2.53) | T(175)=-1.37, p=.17 |
| Males, No. | 31 | 98 | Χ^2^=2.95, p=.13 |
| IQ | 101.15 (11.20) | 102.96 (11.40) | T(175)=-.95, p=.35 |
| ADHD diagnoses, N | 5 | 20 | Χ^2^=.82, p=.47 |
| CBCL externalizing problems, mean (SD) | 16.66 (13.56) | 18.84 (15.26) | T(162)=-.81, p=.42 |
| CBCL conduct problems, mean (SD) | 5.87 (5.81) | 7.37 (6.89) | T(164)=-1.25, p=.22 |
| CBCL rule breaking, mean (SD) | 4.73 (4.48) | 6.29 (6.43) | T(164)=-1.44, p=.09 |
| CBCL aggression, mean (SD) | 11.93 (9.66) | 12.77 (10.02) | T(163)=-.47, p=.64 |
| RPQ proactive, mean (SD) | 3.82 (5.38) | 2.93 (3.65) | T(162)=1.20, p=.32 |
| RPQ reactive, mean (SD) | 10.32 (5.94) | 9.56 (5.15 | T(162)=.80, p=.42 |
| RPQ total, mean (SD) | 14.14 (10.51) | 12.49 (7.90) | T(162)=1.08, p=.28 |
| ICU total, mean (SD) | 30.98 (10.70) | 27.17 (11.02) | T(164)=1.96, p=.051 |

| Table S3. Scanning parameters of the anatomical images across sites. | | | | | | | |
| --- | --- | --- | --- | --- | --- | --- | --- |
| Scanner | Site | TR^*^/TE/TI (ms) | Flip angle | Field of view | Matrix RL/AP/slices | Voxel size (mm) | Acceleration factor |
| Siemens | Nijmegen | 2300/2.98/900 | 9 | 256 | 212/256/176 | 1.0x1.0x1.2 | 2 |
|  | Mannheim | 2300/2.96/900 | 9 | 256 | 212/256/176 | 1.0x1.0x1.2 | 2 |
|  | Barcelona | 2300/2.98/900 | 9 | 256 | 212/256/176 | 1.0x1.0x1.2 | 2 |
|  | Madrid | 2300/2.98/900 | 9 | 256 | 212/256/176 | 1.0x1.0x1.2 | 2 |
| GE | London | 7.31/3.02/400 | 11 | 270 | 256/256/196 | 1.0x1.0x1.2 | 1.75 |

Table S4. Normative models of the anatomical dataset.

| **Model** | **Deviations** | **Participant** | **N** | **Mean rank** | **Sum of ranks** | **Mann-Whitney U** |
| --- | --- | --- | --- | --- | --- | --- |
| Whole sample as reference cohort | Positive | controls | 94 | 134.80 | 12671.50 | 6269.50, *p*=.08 |
|  |  | cases | 154 | 118.21 | 18204.50 |  |
|  | Negative | controls | 94 | 116.51 | 10952.00 | 6487, *p*=.17 |
|  |  | cases | 154 | 129.38 | 19924.00 |  |
| Controls only as reference, cases as test sample | Positive | controls | 94 | 133.04 | 12505.50 | 6435.50, *p*=.14 |
|  |  | cases | 154 | 119.29 | 18370.50 |  |
|  | Negative | controls | 94 | 113.57 | 10676.00 | 6211, *p*=.06 |
|  |  | cases | 154 | 131.17 | 20200.00 |  |

Table S5. Demographics across sites

|  | Controls | Cases |
| --- | --- | --- |
| **Nijmegen (n=43)** | 24 | 19 |
| Age, mean (SD) | 13.37 (2.34) | 13.93 (2.37) |
| Males, No. | 16 | 13 |
| IQ | 107.11 (13.31) | 97.23 (10.19) |
| ADHD diagnoses, N | 0 | 4 |
| CBCL externalizing problems, mean (SD) | 4.79 (4.92) | 26.84 (9.65) |
| CBCL conduct problems, mean (SD) | 1.58 (2.3) | 10.63 (4.49) |
| CBCL rule breaking, mean (SD) | 1.63 (1.97) | 8.26 (4.12) |
| CBCL aggression, mean (SD) | 3.17 (3.31) | 18.58 (6.73) |
| RPQ proactive, mean (SD) | .79 (1.41) | 3.82 (3.41) |
| RPQ reactive, mean (SD) | 6.54 (3.67) | 12.53 (3.91) |
| RPQ total, mean (SD) | 7.33 (4.50) | 16.35 (6.49) |
| ICU total, mean (SD) | 19.25 (6.74) | 27.76 (7.34) |
| **Mannheim (n=32)** | 7 | 25 |
| Age | 13.99 (3.39) | 12.91 (2.73) |
| Sex | 6 | 22 |
| IQ | 116.79 (6.20) | 102.44 (10.02) |
| ADHD diagnoses | 0 | 3 |
| CBCL externalizing problems, mean (SD) | 4.00 (6.66) | 28.75 (12.22) |
| CBCL conduct problems, mean (SD) | 1.71 (2.43) | 12.29 (6.63) |
| CBCL rule breaking, mean (SD) | 1.71 (2.75) | 10.38 (7.16) |
| CBCL aggression, mean (SD) | 2.43 (3.95) | 18.46 (6.55) |
| RPQ proactive, mean (SD) | 1.29 (2.22) | 4.25 (4.52) |
| RPQ reactive, mean (SD) | 7.14 (3.81) | 10.83 (5.57) |
| RPQ total, mean (SD) | 8.43 (5.65) | 15.08 (8.90) |
| ICU total, mean (SD) | 22.71 (7.70) | 34.83 (11.11) |
| **London (n=13)** | 7 | 6 |
| Age | 13.79 (2.12) | 15.03 (2.48) |
| Sex | 5 | 6 |
| IQ | 112.28 (8.53) | 91.35 (11.89) |
| ADHD diagnoses | 0 | 4 |
| CBCL externalizing problems, mean (SD) | 3.86 (5.46) | 22.60 (7.54) |
| CBCL conduct problems, mean (SD) | 1.00 (2.24) | 7.00 (2.83) |
| CBCL rule breaking, mean (SD) | 1.14 (2.61) | 5.40 (2.51) |
| CBCL aggression, mean (SD) | 2.71 (2.98) | 17.20 (5.26) |
| RPQ proactive, mean (SD) | .43 (.79) | 2.50 (2.43) |
| RPQ reactive, mean (SD) | 3.71 (2.69) | 15.17 (2.32) |
| RPQ total, mean (SD) | 4.14 (3.08) | 17.67 (2.50) |
| ICU total, mean (SD) | 21.43 (11.84) | 29.00 (6.42) |
| **Barcelona (n=25)** | 9 | 16 |
| Age | 14.59 (2.44) | 12.71 (2.59) |
| Sex | 4 | 13 |
| IQ | 106.62 (5.27) | 98.96 (9.17) |
| ADHD diagnoses | 0 | 4 |
| CBCL externalizing problems, mean (SD) | 5.11 (4.23) | 32.19 (7.00) |
| CBCL conduct problems, mean (SD) | .78 (1.30) | 11.88 (4.60) |
| CBCL rule breaking, mean (SD) | .78 (1.30) | 10.06 (6.04) |
| CBCL aggression, mean (SD) | 4.33 (3.28) | 22.13 (4.56) |
| RPQ proactive, mean (SD) | 1.67 (2.06) | 3.85 (3.60) |
| RPQ reactive, mean (SD) | 5.89 (3.76) | 12.69 (2.93) |
| RPQ total, mean (SD) | 7.56 (5.50) | 16.54 (5.53) |
| ICU total, mean (SD) | 18.63 (10.13) | 34.13 (12.34) |
| **Madrid (n=16)** | 5 | 11 |
| Age | 15.06 (1.92) | 14.26 (2.32) |
| Sex | 3 | 10 |
| IQ | 102.22 (8.58) | 99.75 (9.19) |
| ADHD diagnoses | 0 | 5 |
| CBCL externalizing problems, mean (SD) | 2.75 (2.06) | 32.75 (19.14) |
| CBCL conduct problems, mean (SD) | 1.00 (1.16) | 13.10 (8.53) |
| CBCL rule breaking, mean (SD) | 1.00 (1.16) | 12.20 (8.07) |
| CBCL aggression, mean (SD) | 1.75 (1.50) | 21.00 (12.84) |
| RPQ proactive, mean (SD) | 1.00 (1.00) | 7.10 (4.53) |
| RPQ reactive, mean (SD) | 3.00 (1.73) | 12.30 (4.40) |
| RPQ total, mean (SD) | 4.00 (1.00) | 19.40 (8.40) |
| ICU total, mean (SD) | 20.50 (6.40) | 31.20 (7.66) |

Note: No site by group effects were found (age: p=.24, sex: p=.11, IQ: p=.82, CBCL externalizing problems: p=.19, CBCL conduct problems: p=.39, CBCL rule breaking: p=.21, CBCL aggression: p=.26, RPQ proactive: p=.49, RPQ reactive: p=.12, RPQ total: p=.18, ICU total: p=.61). No differences across site were found with regard to ADHD (p=.15)

Table S6. Case-control differences regarding deviations negative, positive/neutral face processing and shapes.

| **Modality** | **Participant** | **N** | **Mean rank** | **Sum of ranks** | **Mann-Whitney U** |
| --- | --- | --- | --- | --- | --- |
| Negative faces | controls | 52 | 55.10 | 2865.00 | 1487, *p_FDR_*=.018 |
|  | cases | 77 | 71.69 | 5520.00 |  |
| Positive faces | controls | 52 | 53.22 | 2767.50 | 1389, *p_FDR_* =.009 |
|  | cases | 77 | 72.95 | 5617.50 |  |
| Shapes | controls | 52 | 55.54 | 2888.00 | 1510, *p_FDR_* =.018 |
|  | cases | 77 | 71.39 | 5497.00 |  |
