# Supplementary figures and images for: Age-related brain deviations and aggression
Source: Psychol Med. 2022 Apr 22;53(9):4012–21. doi: 10.1017/S003329172200068X (PMC10325848; doi:10.1017/S003329172200068X)

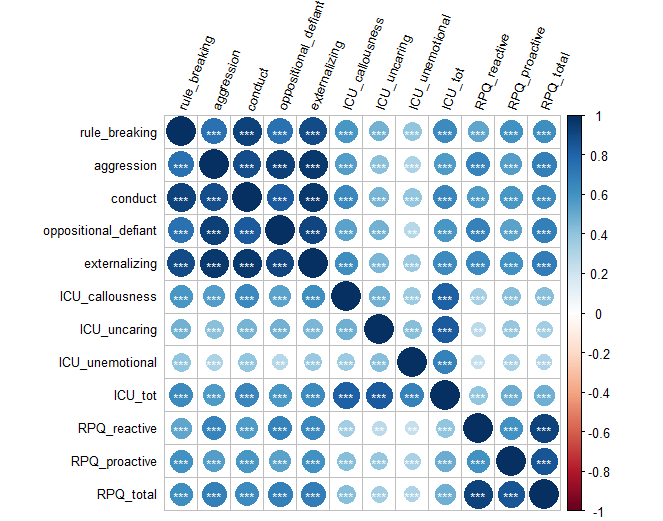

Supplement: Supplementary file 1 [file S003329172200068Xsup.zip › S003329172200068Xsup001.tif]

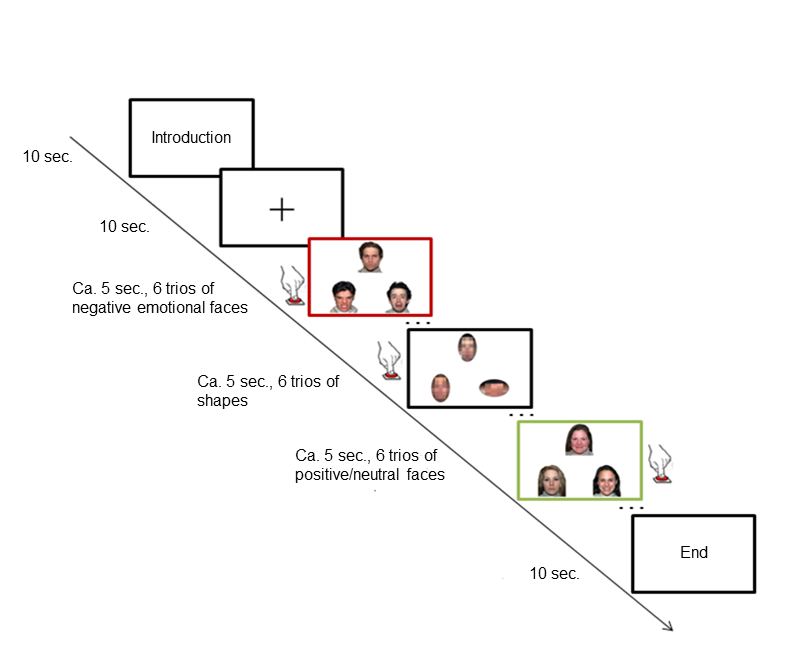

Supplement: Supplementary file 1 [file S003329172200068Xsup.zip › S003329172200068Xsup002.tif]

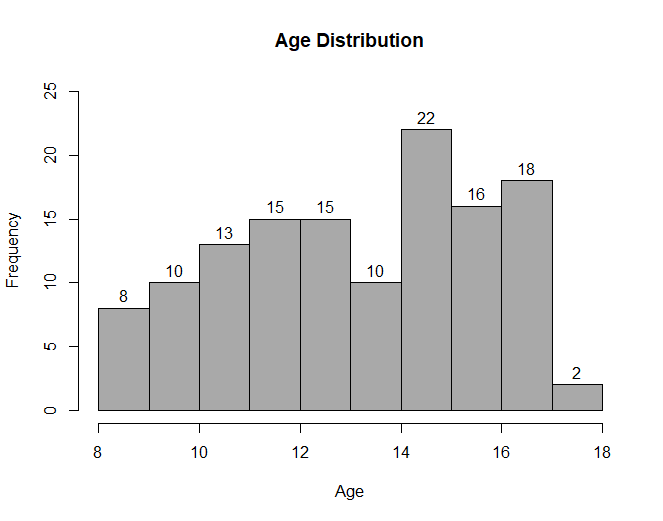

Supplement: Supplementary file 1 [file S003329172200068Xsup.zip › S003329172200068Xsup003.tif]

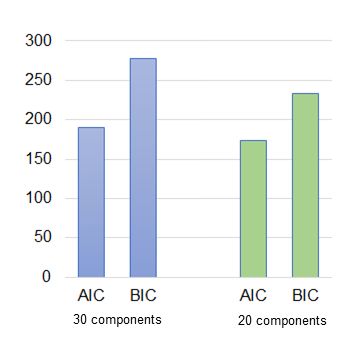

Supplement: Supplementary file 1 [file S003329172200068Xsup.zip › S003329172200068Xsup004.tif]

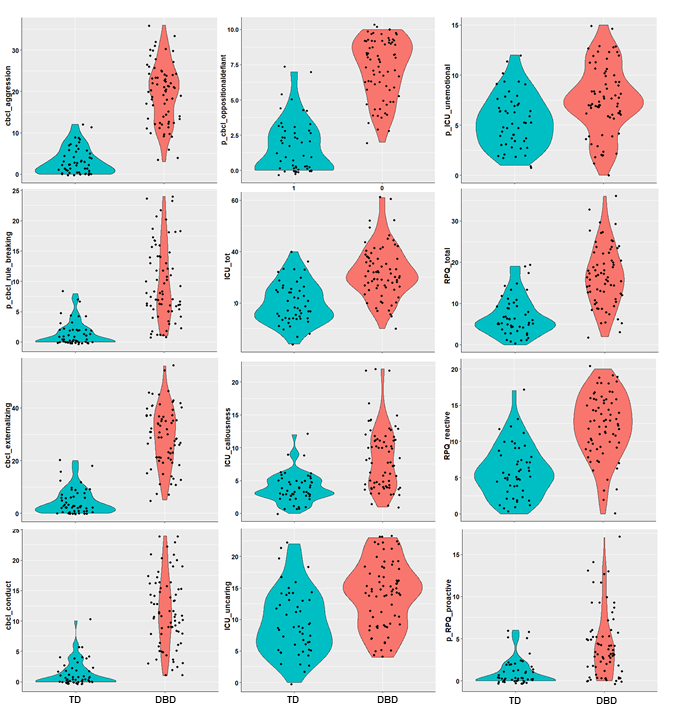

Supplement: Supplementary file 1 [file S003329172200068Xsup.zip › S003329172200068Xsup005.tif]

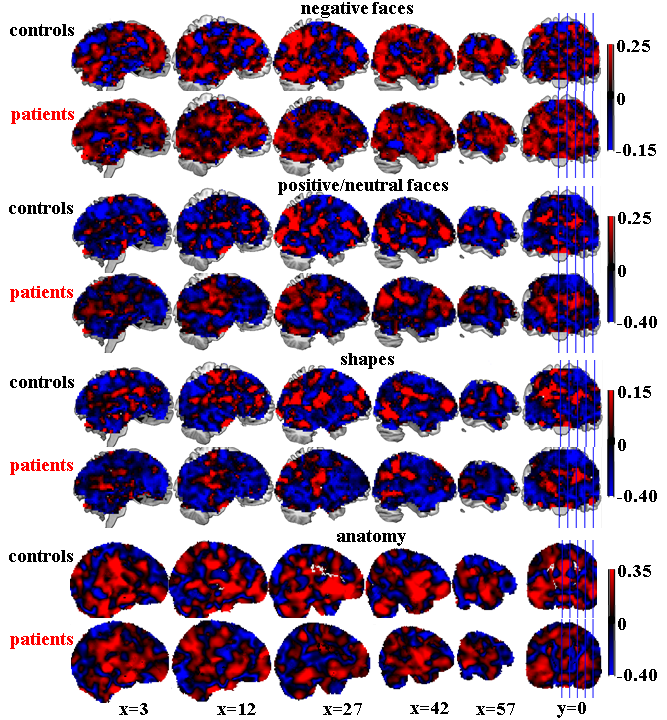

Supplement: Supplementary file 1 [file S003329172200068Xsup.zip › S003329172200068Xsup006.tif]
